# Ecstasy induces reactive oxygen species, kidney water absorption and rhabdomyolysis in normal rats. Effect of N-acetylcysteine and Allopurinol in oxidative stress and muscle fiber damage

**DOI:** 10.1371/journal.pone.0179199

**Published:** 2017-07-05

**Authors:** Ana C. de Bragança, Regina L. M. Moreau, Thales de Brito, Maria H. M. Shimizu, Daniele Canale, Denise A. de Jesus, Ana M. G. Silva, Pedro H. Gois, Antonio C. Seguro, Antonio J. Magaldi

**Affiliations:** 1 Clinical Hospital, School of Medicine-Department of Nephrology- Basic Research Laboratory-LIM12, University of Sâo Paulo, SP, Brazil; 2 School of Pharmaceutical Sciences, Department of Clinical and Toxicological Analysis, University of São Paulo, SP, Brazil; 3 School of Medicine, Institute of Tropical Medicine, Department of Pathology, University of São Paulo, SP, Brazil; 4 School of Medicine, Department of Nephrology, University of Sâo Paulo, SP, Brazil; University of Sao Paulo Medical School, BRAZIL

## Abstract

**Background:**

Ecstasy (Ec) use produces hyperthermia, excessive sweating, intense thirst, an inappropriate antidiuretic hormone secretion (SIADH) and a multisystemic toxicity due to oxidative stress (OS). Intense thirst induces high intake of pure water, which associated with SIADH, usually develops into acute hyponatremia (Hn). As Hn is induced rapidly, experiments to check if Ec acted directly on the Inner Medullary Collecting Ducts (IMCD) of rats were conducted. Rhabdomyolysis and OS were also studied because Ec is known to induce Reactive Oxygen Species (ROS) and tissue damage. To decrease OS, the antioxidant inhibitors N-acetylcysteine (NAC) and Allopurinol (Allo) were used.

**Methods:**

Rats were maintained on a lithium (Li) diet to block the Vasopressin action before Ec innoculation. AQP2 (Aquaporin 2), ENaC (Epitheliun Sodium Channel) and NKCC2 (Sodium, Potassium, 2 Chloride) expression were determined by Western Blot in isolated IMCDs. The TBARS (thiobarbituric acid reactive substances) and GSH (reduced form of Glutathione) were determined in the Ec group (6 rats injected with Ec-10mg/kg), in Ec+NAC groups (NAC 100mg/Kg/bw i.p.) and in Allo+Ec groups (Allo 50mg/Kg/i.p.).

**Results:**

Enhanced AQP2 expression revealed that Ec increased water transporter expression, decreased by Li diet, but the expression of the tubular transporters did not change. The Ec, Ec+NAC and Allo+Ec results showed that Ec increased TBARS and decreased GSH, showing evidence of ROS occurrence, which was protected by NAC and Allo. Rhabdomyolysis was only protected by Allo.

**Conclusion:**

Results showed that Ec induced an increase in AQP2 expression, evidencing another mechanism that might contribute to cause rapid hyponatremia. In addition, they showed that NAC and Allo protected against OS, but only Allo decreased rhabdomyolysis and hyperthermia.

## Introduction

Ecstasy (Ec), or 3,4-methylenedioxymethamphetamine (MDMA), is one of the amphetamine compounds most widely used as a recreational drug of abuse. It is a central nervous system stimulant causing important side effects [1]. Among these, the most prevalent are hyperactivity, hyperpyrexia, profuse sweating, thirst, hyponatremia, rhabdomyolysis, and oxidative stress (OS) [1]. Ec also induces an Antidiuretic Hormone secretion (ADH) [2,3], resulting in an inappropriate antidiuretic hormone secretion (SIADH). This clinical syndrome occurs by a direct action of Ec and by its metabolites such as HHMA (3,4-dihydroxymethamphetamine), HMMA (4 hydroxy-3-methoxymethamphetamine), HMA (4-hydroxy-3-methoxyamphetamine) and MDA (methylenedioxyamphetamine)(1).

One of the most important causes of death is an acute cerebral edema as a consequence of hyponatremia [4,5,6,7]. Hyperthermia, accompanied by intense physical activity in hot environments, causes intense sweating with a high loss of water and sodium thus producing hypertonic dehydration. The extracellular volume contraction simultaneously stimulates thirst and ADH delivery leading to a profuse consumption of water without salt ingestion and an increase in water absorption by the inner medullary collecting duct (IMCD). These processes are intensified by the SIADH, accelerating these mechanisms. This unrestricted and abundant water ingestion produces rapid hydric intoxication, causing hyponatremia and low plasma osmolality. This low osmolality induces cerebral swelling (edema) which causes neurologic complications, such as seizures, brain-stem herniation through the foramen magnum, coma, respiratory arrest and death [4,7].

Acute and sub-chronic use of Ec increases OS, thus contributing to tissue injury in many organs and in some cases to death [8,9,10]. There is some evidence that hyperpyrexia along with high room temperature [11,12] are important conditions that increase the toxic effect of Ec, the development of the ROS and OS. The peak of Ec concentration in blood occurs 2h circa after its ingestion, and it is metabolized in the liver [9,10,13].

It has already been reported that anti-oxidant compounds are able to attenuate the OS produced by Ec. One of them is N-acetylcysteine (NAC), which is a pro-drug of L-cysteine and a precursor of the biologic antioxidant GSH [14]. Another drug used is Allopurinol (Allo), an inhibitor of the xanthine-oxidase, which is employed as the main therapeutic agent to block the hyper production of uric acid in cases of gout. However, Allo also has a potent anti-oxidant effect since it blocks that particular enzyme. Thus, it has been used not only to treat gout but also as an anti-oxidant drug [15,16,17].

Another important finding resulting from Ec use is acute kidney injury (AKI) due to myoglobin deposition in the kidney caused by rhabdomyolysis.

Rhabdomyolysis is a complex syndrome with a multifactorial etiology [18,19]. It is characterized by a breakdown of muscle cells, followed by diffusion of their intracellular components, such as, potassium, myoglobin, creatine kinase (CK) into the blood and extracellular space. It can be caused by traumas, infections, muscle ischemia, intense muscular exercise, thermo-dependent syndromes, medicines, toxins, seizures and immobility, illicit drugs, alcohol abuse and electrolyte imbalance. The classic triad of symptoms of rhabdomyolysis consists of myalgia, weakness and tea-colored urine. [20,21].

The aims of this study were to evaluate the effect of Ec on renal function, considering the possibility that it acts directly in the IMCD, increasing water reabsorption. The authors also investigated whether or not antioxidants could prevent OS and rhabdomyolysis.

## Materials and methods

### Animals

Male Wistar rats, weighing 170-180g, were obtained from the animal facilities of the University of São Paulo Medicine School. They were kept under standard laboratory conditions and fed a normal pellet diet and tap water *ad libitum*. The project, experiments and euthanasia procedure, was approved by the Ethics Committee of the Clinical Hospital of the University of São Paulo Medical School.

### Clearance of inulin

To study renal filtration, rats received. 10mg/Kg of Ec i.p. diluted in 1ml of saline solution. After one hour, when the rats were agitated, sweating profusely with bristling fur and eyes wide–open, they were prepared for the clearance study to determine the Glomerular Filtration Rate (GFR). They were anesthetized with sodium thiopental (i.p.- 50 mg/kg body weight). The trachea was always cannulated with a polyethylene (PE-240) catheter to facilitate spontaneous breathing. The control group was inoculated with 1ml of saline solution.

A constant infusion of inulin (10 mg/kg body weight in 0.9% saline) was administered at 0.04 mL/min throughout the experiment. Three urine samples were collected at 30-minute intervals (4 samples/animal). Blood samples were obtained at the beginning and at the end of the experiment. Blood and urine inulin were determined using the anthrone method [22].

### Cellular transporter evaluation

To study the effect of Ec in water and sodium transporters along the nephrons, the rats were inoculated one hour previously with Ec (10 mg/kg bw i.p.), and when they had the symptoms described above, they were euthanized.

To study the Ec effect on water transporter Aquaporin 2 (AQP2) in the IMCD cells without the effect of ADH, the authors opted to feed the rats a lithium diet (40 mmol/kg/food, for 5 days) before determining the AQP2 expression, since this amphetamine induces ADH secretion [2,3].

### Western blot: Preparation of membrane fractions

Renal medulla samples were homogenized in cold isolation solution (20 mM Mannitol, 80 mM Hepes, 41 mM KOH, pH 7.5) containing protease inhibitors (cocktail protease inhibitor, Sigma Chemical, St. Louis, MO, USA) using a Teflon pestle glass homogenizer (Schmidt and Co, Frankfurt/M, Germany) [23]. The homogenates were centrifuged at low speed (4000 g) for 15 min at 4°C in order to remove the nuclei and cell debris. Subsequently, the supernatants were centrifuged at 200,0 g for 1 h at 4°C (rotor 50Ti; Beckman Instruments, Palo Alto, CA, USA) to produce a pellet containing membrane fractions enriched with plasma membranes and intracellular vesicles. Protein concentration was determined for each sample using the Bradford method (Bio-Rad Laboratories, Richmond, CA, USA).

### Electrophoresis and immunoblotting

Proteins were separated on denaturing SDS (sodium dodecyl sulphate) 12% polyacrylamide minigels (for AQP2), 10% polyacrylamide (for ENaC), and 8% polyacrylamide (for NKCC2) by electrophoresis [23]. Proteins were then transferred to a polyvinylidene difluoride (PVDF) membrane by wet electroblotting for 90 min. Blots were blocked for 60 min at 4°C with 5% nonfat dry milk in PBS-T, pH 7.5 (phosphate-buffered saline, in mM: 100 NaCl, 80 Na_2_HPO_4_, 20 NaH_2_PO_4_, 0.1% Tween 20). After that, blots were incubated with the AQP2 antibody (1:2,000 dilution), NKCC2 antibody (0.12 μg/ml), ENaC antibody (1:500), and with the control actin antibody (1:2,000) overnight. Subsequently, they were washed and incubated with the second antibody (anti-goat secondary antibody HRP-conjugated, diluted at 1:10,000) for 1 h. The labeling was visualized with a horseradish peroxidase-conjugated secondary antibody (anti-rabbit IgG, diluted 1:2,000, anti-goat IgG, diluted 1:10,000, or anti-mouse IgG diluted 1:2,000; Sigma), using the enhanced chemiluminescence (ECL) detection system (Amersham Pharmacia Biotech, Piscataway, NJ).

### Quantification of AQP2 kidney levels and sodium transporters

Enhanced chemiluminescence films presenting bands within the linear range were scanned using the Image Master VDS (USA). For AQP2, both the 29kDa and the 35-50kDa bands (corresponding to two different states of glycosylation) were quantified by densitometric analysis. Densitometry results are reported as integrated values (area x density of the band), expressed in percentages and compared to control actin protein abundance (100%).

### Redox state evaluation

Oxidative stress was studied by two different methodologies:1- TBARS- Thiobarbituric acid reactive substances Serum levels of thiobarbituric acid reactive substances (TBARS), which are markers of lipid peroxidation, were determined using thiobarbituric acid assay [24]. Briefly, an 0.2-ml serum sample was diluted in 0.8 ml of distilled water. Immediately thereafter, 1 ml of 17.5% trichloroacetic acid was added. Following the addition of 1 ml of 0.6% thiobarbituric acid, pH 2, the sample was placed in a boiling water bath for 15 min, and then left to cool. Subsequently, 1 ml of 70% trichloroacetic acid was added, and the mixture was incubated for 20 min. The sample was then centrifuged for 15 min at 2,000 rpm. The optical density of the supernatant was read at 534 nm against a reagent blank using a spectrophotometer. The quantity of TBARS was calculated using a molar extinction coefficient of 1.56 × 10^5^ M^− 1^ cm^− 1^. Serum levels of TBARS are expressed as nmol/ml [24]. 2- GSH- Reduced gluthatione (GSH) level in whole-blood was determined by Sediak and Linday methods. To the whole-blood, four volumes of ice-cold 5% (W/V) metaphosphoric acid (MPA) were added and then centrifuged at 14,000g for 3min. Elman’s reagent was added to the supernatant and the yellow pigment formed was measured in a spectrophotometer at 412nm wavelengths. The GSH was quantified using the cysteine standard curve and expressed in μmol/ml [25].

### Rhabdomyolysis

To evaluate the effect of NAC and Allo for preventing or treating rhabdomyolysis, samples of blood and gastrocnemius muscles were collected at the moment of euthanasia. Plasma levels of TBARS, GSH, K^+^ and CK were dosed, and muscles were prepared for histological analysis by hematoxilin-eosin staining (HE). Body temperature was also measured.

The Ec dilution used was 40mg/Kg/bw. This dose was chosen because it can cause an effective increase in hyperthermia [9].

Five groups were prepared for this study: Sham, Ec, Ec+NAC, NAC+E and Ec+Allo. NAC (100mg/Kg/bw) was inoculated i.p. before or after Ec, depending on the group. The i.p. administration was always chosen to reproduce the acute use of the drug. Allo was also injected i.p. 50 mg/Kg/bw after Ec.

Rectal temperature was measured by using a digital thermometer, after one hour of Ec injection, just before euthanasia.

### Drugs

From Ec tablets, provided by the Brazilian Federal Police Department (São Paulo-Brazil), the amphetamine was extracted and purified to 99% at the College of Pharmaceutical Science at the University of São Paulo, Brazil. The final product was lyophilized and then dissolved for use in 0.9% NaCl solution at a concentration of 10 mg/kg or 40mg/kg.

### Statistics

Differences among the means of multiple parameters were analyzed by ANOVA followed by the Student-Newman-Keuls test. Differences between two parameters were analyzed by either paired *t*-test or by nonparametric methods (Mann-Whitney test). Values of p<0.05 were considered significant.

### Ethics statement

This research (project number 048/14 and research number 5519) was approved by the Ethical Committee of animal use of the Medical School of the University of São Paulo-CAPPesq-FMUSP. All animal procedures were conducted following the rules issued by the Brazilian National Council for Control of Animal Experimentation (CONCEA) including euthanasia, by a lethal overdose of sodium thiopental after completion of experiments.

## Results

All animals were maintained in a cage with water *ad libitum*, and one hour after Ec i.p injection, when they presented the aforementioned symptoms, they were studied.

### Clearance study-inulin clearance

In this group, 6 normal rats inoculated with Ec (10 mg/kg bw i.p.) and 8 normal rats inoculated with saline were studied. The results showed that Ec did not change the GFR (ml/min): control 0.854±0.10 and Ec 0.943±0.05 (NS), evidencing that this drug did not alter the filtration rate.

### Analysis of expression of AQP2 by Western blot

**Fig 1:**
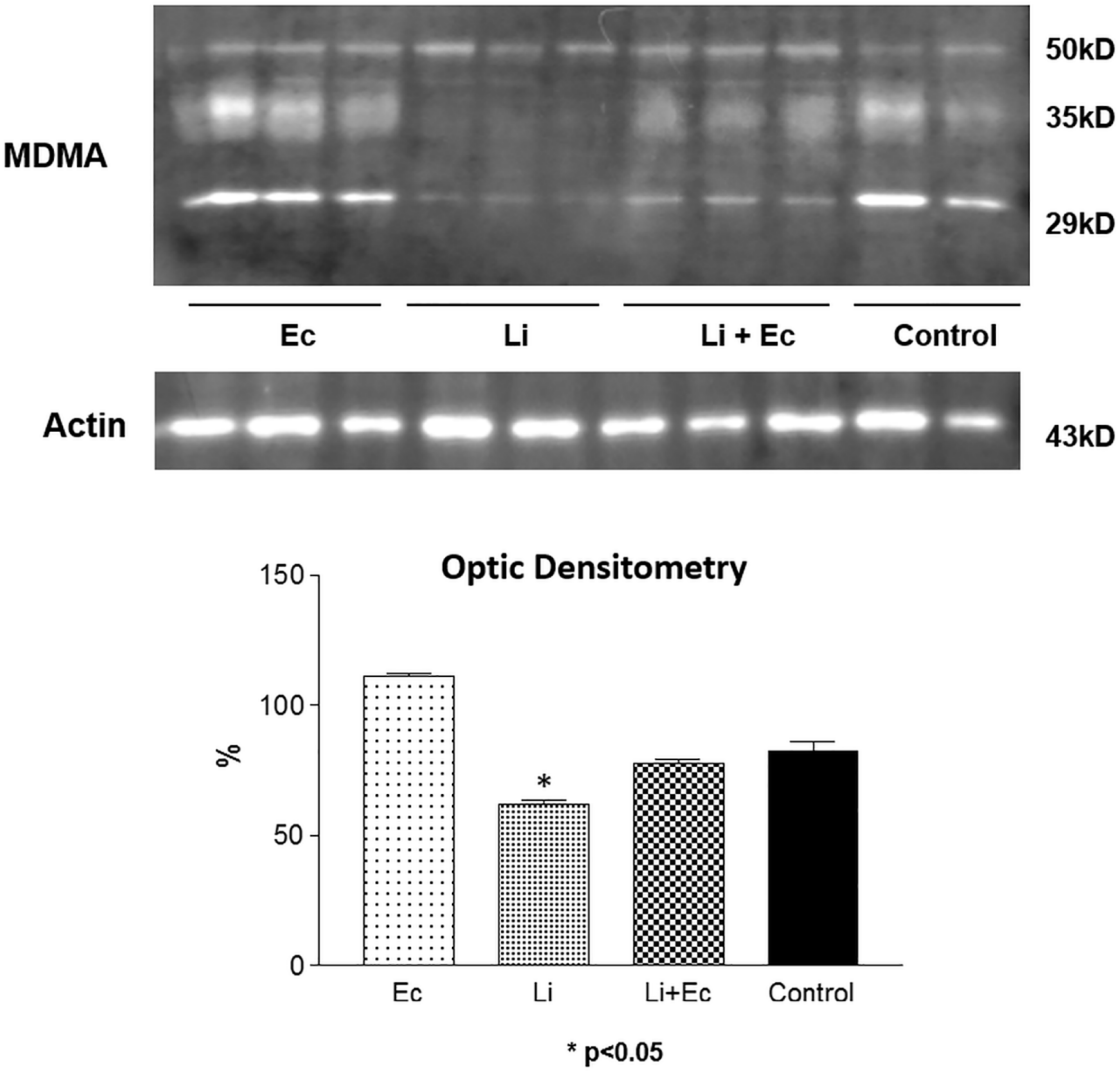
Western-blot analysis of the water transporter. Bands 29 and 35-50kDa from AQP2. * p< 0.05 Li x Li+Ec.

In order to investigate if Ec would be able to increase AQP2 in the absence of ADH, the hormone effect was blocked by lithium pretreatment since this Ec is able to induce an SIADH (2,3). The Western Blot experiments demonstrated that the 29 kDa and the 35–50 kDa bands were increased, corresponding to the nonglycosilated and glycosilated AQP2, respectively. The AQP2 expression, analyzed by optical densitometry of the Western Blot bands, revealed that Ec was able to increase the water transporter expression decreased by lithium therapy (absence of ADH action) as follows: control-100.00±5.08, Li- 68.28±2.39, Li+Ec- 76.40±0.80 p< 0.05 (Fig 1). These results showed that the AQP2 abundance was decreased in the presence of lithium but increased in the presence of Li+Ec, showing a proper effect of this drug on the IMCD, which intensifies the water absorption by this nephron segment.

### Analysis of the expression of membrane transporter and channel

One ion transporter (NKCC2) and one channel (ENaC) along the nephron were studied since they are involved in water absorption. None of them revealed differences in the presence of Ec, compared to the control (Table 1).

**Table 1:** Values of optic densitometry.

	NKCC2	ENaC
Control	100.00±10.78	101.34±12.11
Ec	112.40±17.85	80.99±9.86

n = 5 for each groups; Cont vs Ec- NS for all groups

### Oxidative stress studies

In order to study the efficacy of anti-oxidant agents to prevent or to treat the ROS induced by Ec (10mg/kg bw i.p), NAC was administered before or after amphetamine inoculation. The values of TBARS and GSH are shown in Table 2.

**Table 2:** Values of TBARS and GSH.

	Cont	Ec	NAC+Ec	Ec+NAC
GSH	2.7±0.16	1.89±0.10**	3.04±0.12^+^	2.52±0.40**
TBARS	1.92±0.18	2.56±0.11**	1.83±0.19*	1.38±0.16*
n				

TBARS in nmol/ml; GSH in μmol/ml

*p < 0.001 vs Ec

^+^p < 0.001 vs Ec

**p < 0.05 vs Cont

These results revealed that the NAC is able to decrease the OS induced by Ec when administered before or after this drug (Ec) had been inoculated.

### Rhabdomyolysis

Rhabdomyolysis is an important finding from Ec use [1]. In order to investigate if this muscle injury can be decreased by the use of antioxidant agents, five groups of experiments were carried out and the effects of NAC and Allo were studied. The dose of 40mg/Kg/bw was chosen to induce an effective increase in hyperthermia [9]. Creatine kinase (CK), uric acid (Ur.Ac), sodium (Na^+^), potassium (K^+^), TBARS, GSH plasma levels and body temperature in all groups were measured. The results are in Table 3.

**Table 3:** Values of CK, Ur.Ac, Na^+^, K^+^, TBARS, GSH ant body temperature (T).

	CK	Ur.Ac.	Na^+^	K^+^	TBARS	GSH	T
Sham	382±70	1.5±0.30	143.0±0.02	4.1±0.10	0.60±0.10	2.16±0.08	36.8±0.10
Ec	1473±22^a^	2.03±0.26	141.0±0.02	7.8±0.49	2.31±0.39	2.16±0.08	36.8±0.10
Ec+NAC	2355±48	2.80±0.06	139.3±2.5	8.60±0.10	0.89±0.10*	1.55±0.10*	39.4±1.05
NAC+Ec	2854±98	2.83±0.70	142.0±1.1	7.65±1.65	0.73±0.13*	1.49±0.10*	40.7±0.25
Ec+Allo	935±81**	0.28±0.05**	142.0±1.4	4.75±0.16**	1.50±0.18	2.30±0.13^+^	36.3±0.09^+^

CK- U/L; K^+^ mEq/L; UrAc mg/dL; Temperature in C°

^a^ p<0.01 vs Sham

*p<0.02 vs Ec

**p<0.05 vs Ec

^+^p<0.001 vs Ec; for Ec n = 7; for others n = 4; for sham n = 6

This table shows that NAC did not protect the muscle degeneration caused by Ec since the plasma levels of CK, K^+^ and Uric Acid remained high after the use of this anti-oxidant agent. On the other hand, NAC and Allo protected against ROS. The sodium plasma level did not change in all groups. Body temperature in NAC groups remained high, but was normal in the Allo group.

**Fig 2:**
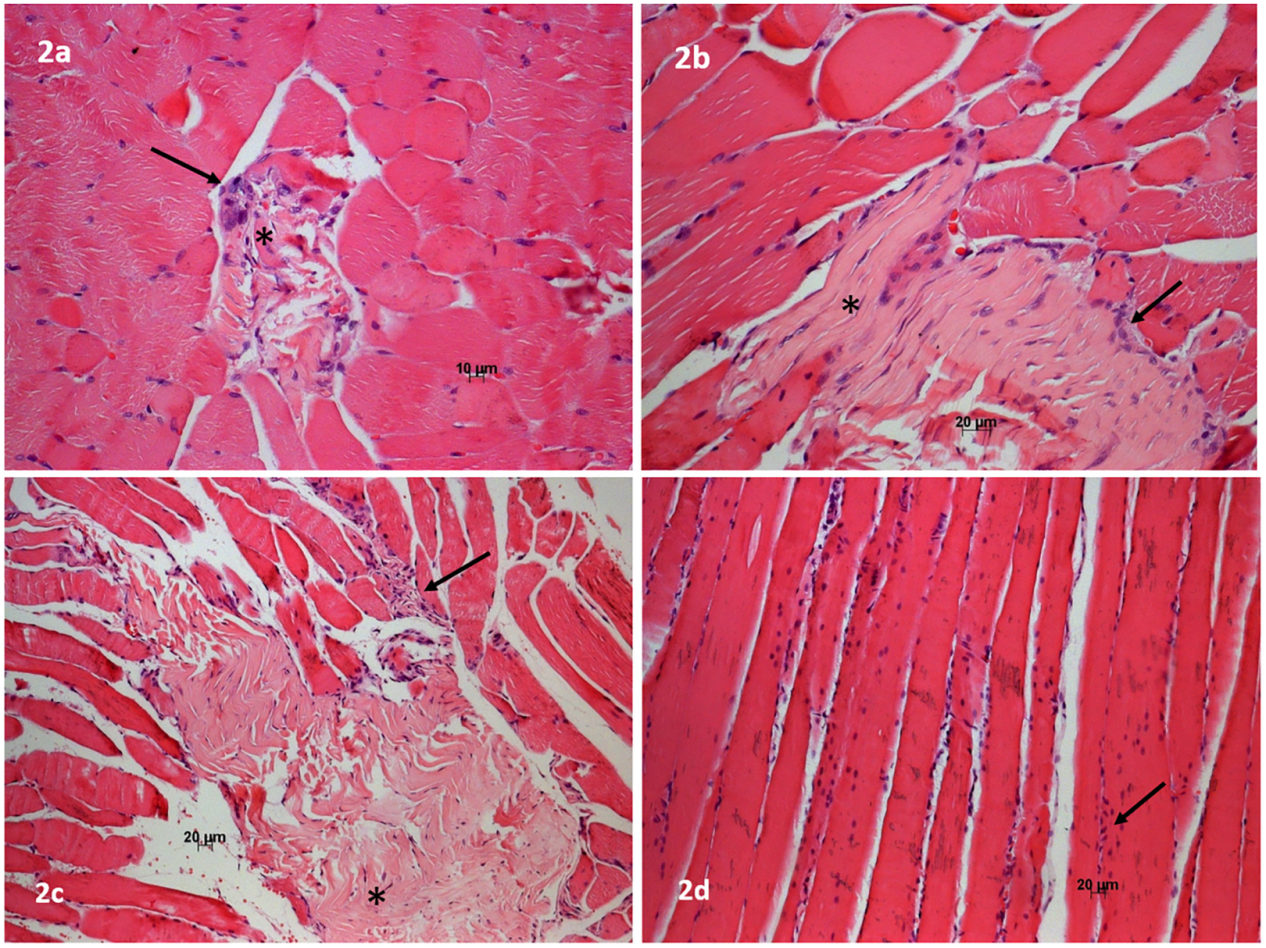
Representative light micrographs of Hematoxylin-Eosin stain. 2a (Ec), 2b (Ec+NAC) and 2c (NAC+Ec) show areas of necrosis (*) and inflammatory infiltrations (macrophages)(arrows). 2d (Allo+Ec) shows no necrosis and slight inflammatory infiltration.

Fig 2A shows that in muscle fragments from Ec groups (control), there are disruptions in muscle fibers, along with focal necrosis in some parts of the fragment and also macrophage infiltrations. Fig 2B and 2C show that NAC was not able to protect the muscle from the Ec action. However, Fig 2 shows there was no muscle damage, and a very slight macrophage infiltrations when Allo was administered.

## Discussion

Our results showed the Ec effect that occurred 60 minutes after its administration in rats. This time was chosen to simulate the high Ec ingestion that usually occurs at raves and parties, which in many cases, induces severe alterations in hydroelectrolyte metabolism and tissue damage, causing high lethality [26,27,28,29,30].

Clearance showed no alteration, compared with normal rats, evidencing that this drug did not modify the GFR during the time of the experiments. There are some references about kidney dysfunctions induced by Ec and its metabolites, but all of them concluded that this is a result of extra-kidney events, principally rhabdomyolysis. [30].

Most fatal cases are due to a cerebral edema induced by hyponatremia that developed rapidly. This hyponatremia is caused both by rapid and intense water ingestion without solute intake and increased water absorption by the kidney. It is noteworthy that the SIADH helps to exacerbate this water absorption [2,3]. Our research did not show a reduction in sodium plasma levels, after Ec inoculation, because the time of the experiments was not enough to decrease them. Hypernatremia, in turn, is a product of an extracellular volume depression caused by intense sweating as a result of a hyperthermia, prolonged exertion and euphoria. Thus, this cascade of events starts with hyperthermia and ends with severe hyponatremia, which can induce cerebral edema. As this hydroelectrolytic disorder occurs very quickly, the possibility of a direct action of Ec on the IMCD cells was considered. To investigate if this hypothesis could be true, blocking the ADH action in the IMCD cells was required, which was done using lithium. In these experiments, in the absence of ADH action, the expression of AQP2 was shown to be increased in the presence of Ec, evidencing that this drug is acting directly in the IMCD cells. This fact evidenced that there might be another mechanism contributing to inducing hyponatremia rapidly.

Other membrane structures, i.e., NKCC2 and EnaC, each one representing one nephron segment, (the thick ascending limb and the distal tubule respectively), were also studied. From these studies, it became clear that Ec or its metabolites did not alter their expression. In contrast, Carvalho et al [31] showed, in an *in-vitro* study, that only Ec metabolites were able to kill proximal tubular cells, and Kwon et al [32] published a case report suggesting a transient proximal tubular renal injury after Ec ingestion. These differences from our data can be explained by the different experimental approaches, by the different nephron segments studied, and also because our data were obtained one hour after Ec administration, differently from their timelines e.

The OS caused by Ec is well known and well documented [33,34]; however, there is not much research about the use of NAC and Allo in these cases [14,35]. Our data showed that these two agents were able to decrease OS (Tables 2 and 3) measured by TBARS and GSH.

The use of antioxidant agents to decrease the rhabdomyolysis produced by Ec is not common and is poorly investigated [36,37 38]. Our results showed that in all groups treated with NAC, the high body temperature did not decrease, but in the group treated with Allo, the temperature did not increase, remaining normal. There are several causes of rhabdomyolysis [14, 39, 40]; nevertheless, there is a consensus that hyperpyrexia, hyperactivity and high environment temperatures are important factors. Particularly in our experimental models, it is possible to consider that only hyperthermia was the principal cause of muscle alteration since the rats were in a cage at normal temperature and engaged in little activity. However, the high room temperature is an adjuvant cause contributing to high body temperature in Ec users [11,14]. These factors coupled with the short time after Ec inoculation, which was not enough to produce muscle lesions detectable by histological examination, can explain why the muscle damage was not so intense (Fig 2). Diaz et al [41] and recently, Katz et al [42], showed that the effect of Nac to protect the muscle from tetanic fatigue decreased at high temperatures (~40°C). This fact could help explain why Nac did not act during Ec hyperthermia, but these mechanisms remain unclear to date. As the temperature did not increase in the Allo experiments, it would be possible to suppose that this could be one of the causes accounting for the protection provided by the xanthine-oxidase inhibitor, since hyperpyrexia is an important factor for muscle degeneration [11]. It is also possible to infer that the Allo anti-inflammatory properties [43, 44] can explain why this muscle inflammatory infiltration was very slight when this drug was used.

In conclusion, our data showed that Ec was able to directly stimulate the increase of AQP2 in IMCD cells, evidencing, for the first time, the existence of another mechanism that intensifies water absorption quickly, increasing the hyponatremia. Moreover, the use of NAC and Allo decreased the OS equally; however, only Allo was able to block or, at least, to retard the muscle degeneration and the occurrence of rhabdomyolysis. The use of Allo could offer a new approach in the treatment of the consequences of Ec abuse, offering new possibilities to decrease the hyperthermia and rhabdomyolysis thus preventing acute renal injury and decreasing inflammatory infiltration.

## Supporting information

S1 File Clearance data, Control vs Ecstasy- NS; AQP2 expression densitometry- p< 0.05 Li x Li+Ec. (DOCX)
