# Correction: Ecstasy induces reactive oxygen species, kidney water absorption and rhabdomyolysis in normal rats. Effect of N-acetylcysteine and Allopurinol in oxidative stress and muscle fiber damage

**DOI:** 10.1371/journal.pone.0192535

**Published:** 2018-02-01

**Authors:** Ana C. de Bragança, Regina L. M. Moreau, Thales de Brito, Maria H. M. Shimizu, Daniele Canale, Denise A. de Jesus, Ana M. G. Silva, Pedro H. Gois, Antonio C. Seguro, Antonio J. Magaldi

There is an error in the last sentence of the Results section. The correct sentence is: However, Fig 2D shows there was no muscle damage, and a very slight macrophage infiltrations when Allo was administered.

In Table 3, the values in the Ec row under columns GSH and T are incorrect. Please see the corrected Table 3 here.

**Table 3:** Values of CK, Ur.Ac., Na+, K+, TBARS, GSH and body temperature (T).

	CK	Ur.Ac.	Na^+^	K^+^	TBARS	GSH	T
Sham	382±70	1.5±0.30	143.0±0.02	4.1±0.10	0.60±0.10	2.16±0.08	36.8±0.10
Ec	1473±22^a^	2.03±0.26	141.0±0.02	7.8±0.49	2.31±0.39	0.88±0.16	40.3±0.18
Ec+NAC	2355±48	2.80±0.06	139.3±2.5	8.60±0.10	0.89±0.10*	1.55±0.10*	39.4±1.05
NAC+Ec	2854±98	2.83±0.70	142.0±1.1	7.65±1.65	0.73±0.13*	1.49±0.10*	40.7±0.25
Ec+Allo	935±81**	0.28±0.05**	142.0±1.4	4.75±0.16**	1.50±0.18	2.30±0.13^+^	36.3±0.09^+^

CK- U/L; K^+^ mEq/L; UrAc mg/dL; Temperature in C°;

^a^ p<0.01 vs Sham

*p<0.02 vs Ec;

**p<0.05 vs Ec;

^+^p<0.001 vs Ec; for Ec n = 7; for others n = 4; for sham n = 6
