# Supplementary material for: Ecstasy induces reactive oxygen species, kidney water absorption and rhabdomyolysis in normal rats. Effect of N-acetylcysteine and Allopurinol in oxidative stress and muscle fiber damage
Source: PLoS One. 2017 Jul 5;12(7):e0179199. doi: 10.1371/journal.pone.0179199 (PMC5497951; doi:10.1371/journal.pone.0179199)
Supplement: S1 File — (DOCX) [file pone.0179199.s001.docx]

**Data**

**Ref.- PONE-D-16-51417- Ecstasy induces reactive oxygen species, kidney water absorption and rhabdomyolysis in normal rats. Effect of N- acetylcysteine and Allopurinol in oxidative stress and muscle fiber damage**

**GFR (ml/min): control 0.854±0.10 and Ec 0.943±0.05**

**AQP2: : control-100.00±5.08, Li- 68.28±2.39, Li+Ec- 76.40±0.80 p< 0.05**

**Clearance data**

|  | **Control** | **Ecstasy** |
| --- | --- | --- |
| 1 | 0.749 | 1.155 |
| 2 | 0.884 | 1.018 |
| 3 | 0.958 | 0.632 |
| 4 | 0.971 | 0.911 |
| 5 | 0.888 | 0.866 |
| 6 | 0.887 | 0.542 |
| 7 | 1.196 |  |
| 8 | 1.011 |  |
| **x+SE** | **0.943±0.05** | **0.854±0.10** |

**Control vs Ecstasy- NS**

**AQP2 expression-densitometry**

|  | **Control** | **Lithium** | **Lithium+Ecstasy** | **Ecstasy** |
| --- | --- | --- | --- | --- |
|  | 102.7 | 63.88 | 75.05 | 109.14 |
|  | 97.3 | 69.68 | 77.15 | 112.35 |
|  |  | 71.28 | 77.02 | 112.29 |
|  |  |  |  |  |
| **x+SE** | **100.00±5.08** | **68.28±2.39** | **76.40±0.80** | **111.26±1.10** |
|  |  |  |  |  |

**p< 0.05 Li x Li+Ec.**
